# Antibacterial Mechanism of Rhamnolipids against *Bacillus cereus* and Its Application in Fresh Wet Noodles

**DOI:** 10.3390/molecules28196946

**Published:** 2023-10-06

**Authors:** Yongwu Niu, Yiming Sun, Yanxiao Yang, Ben Niu, Yuchen Wang, Shan Qiao

**Affiliations:** 1 National Engineering Research Center for Wheat and Corn Further Processing, Henan University of Technology, Zhengzhou 450001, China; 2 College of Food Science and Engineering, Henan University of Technology, Zhengzhou 450001, China

**Keywords:** rhamnolipids, *Bacillus cereus*, antibacterial activity, fresh wet noodles

## Abstract

*Bacillus cereus* (*B. cereus*) is a common foodborne pathogen causing food poisoning incidents. This study aimed to evaluate the antibacterial activity and underlying mechanism of rhamnolipids (RLs) against *B. cereus*. The minimum inhibitory concentration (MIC) and minimum bactericidal concentration (MBC) of RLs for *B. cereus* were determined to be 16.0 mg/L and 32.0 mg/L, respectively. Scanning electron microscopy and fluorescence microscope images, as well as data of membrane potential, relative electric conductivity, and leakage of intracellular components revealed that RLs disrupted the integrity of the cell membrane. Furthermore, the reactive oxygen species content, catalase (CAT) and superoxide dismutase (SOD) activity indicated that RLs activated the oxidative stress response of *B. cereus* in response to RLs. Fresh wet noodles (FWN) were used as a food model, and RLs showed a significant killing effect on *B. cereus* with a sustained inhibitory effect at the concentrations ranging from 128.0 to 1024.0 mg/kg. Additionally, RLs promoted the conversion of free water to bound water in FWN, which improved the storage of FWN and made the taste more resilient and chewy. These results suggest that RLs could be a potential alternative to antimicrobial agents and preservatives for applications in food processing.

## 1. Introduction

In recent years, there have been vast amounts of economic and social losses due to food spoilage caused by foodborne pathogens, thus garnering continuous attention from the global public health sector [1]. *Bacillus cereus* (*B. cereus*), a Gram-positive bacterium commonly found in the environment, such as air and soil, is the fifth leading cause of food-borne illnesses worldwide, accounting for 1.4%–12.0% of all food borne illnesses, following *Salmonella*, *Campylobacter*, *Norovirus*, and *Staphylococcus aureus* [2,3].

Researches have revealed that *B. cereus* is easily able to contaminate food stuffs with high starch content, such as noodles and rice [4]. Takabe et al. discovered an incident of food poisoning that affected 50 people due to noodles contaminated with *B. cereus* [5]. Wijnands et al. conducted a study on instant noodles in four cafeterias, which demonstrated that 88% of them contained pathogenic levels of *B. cereus* [6]. Consequently, there is a continuous demand for the exploration and development of safe and effective methods to reduce, kill, or eliminate *B. cereus* in food items.

Rhamnolipids (RLs), a type of glycolipid biosurfactant, have been observed to possess various properties and physiological activities, including antibacterial, emulsifying and antiviral abilities [7,8,9]. *Pseudomonas* strains are known to be the most efficient producers of glycolipids that contain rhamnose and 3-hydroxy fatty acids. Particularly, *Pseudomonas aeruginosa* has been identified as the most suitable microorganism for producing two classes of rhamnolipids, mono-rhamnolipids and di-rhamnolipids, with remarkable surface activity [10,11]. In 2003 and 2004, the United States Federal Register published two documents on its official website, 68 FR 25,026 and 68 FR 16,796, which detail the application and approval for permissible levels of rhamnolipid biosurfactants in food by the Environmental Protection Agency. Currently, RLs are used to enhance dough texture, stability, volume and conservation of baked foods such as bread and cake [12,13,14]. However, there are no data on its usage in FWN and other food stuffs. 

Findings from research suggest that the use of RLs is very effective in inhibiting foodborne pathogens. De Rienzo et al. observed that RLs concentrations higher than 0.5% (*v/v*) can restrict the proliferation of *Staphylococcus aureus* [15]. Magalhães et al. discovered that 78.1 mg/mL RLs can suppress the growth of *Listeria monocytogenes* [16]. In 2019, Ferreira established that RLs can significantly impede the vegetative cell state of *B. cereus*, but did not delve into the underlying antibacterial mechanism [17]. Bertuso et al. showed that RLs inhibited the growth and spore germination of *B. cereus*, augmenting the antimicrobial potency of celery oleoresin and limonene compounds tested [18]. Furthermore, Bertuso et al. demonstrated that RLs had an equivalent antibacterial impact on *B. cereus* vegetative cells and endospores as that observed in skim milk [19]. 

In this context, the present research aimed to evaluate the antibacterial activity of RLs against *B. cereus* in FWN and its effect on food quality of FWN, thereby laying the groundwork for the wider application of RLs in FWN and other food industries. Based on these, the oxford cup experiment, scanning electron microscope and fluorescence inverted microscope were employed to investigate the antibacterial effect of RLs on *B. cereus*. To uncover the antibacterial mechanism, the indicators such as cell membrane integrity, permeability, leakage of intracellular macromolecules, and the balance of intracellular redox reactions before and after RLs treatment were characterized.

## 2. Results and Discussion

### 2.1. Effects of pH on the Growth of B. cereus

**Figure 1:**
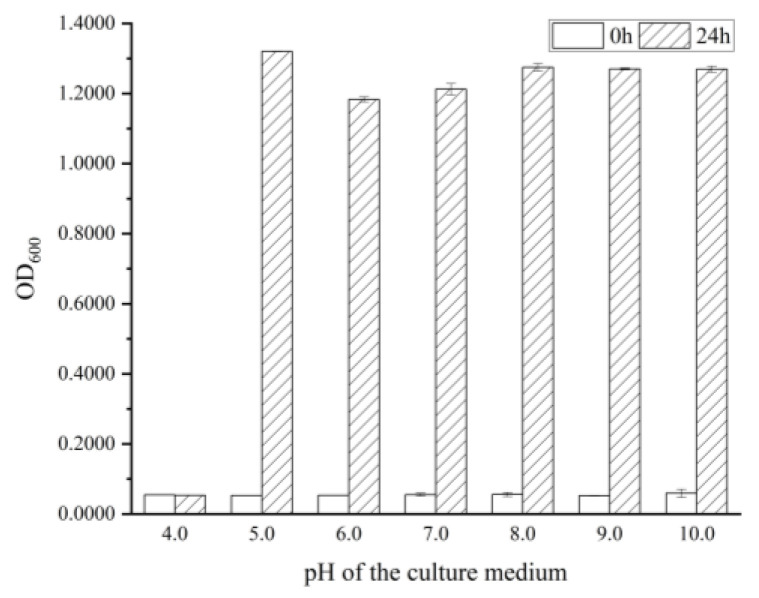
The growth of *B. cereus* at different initial pH values.

Previous studies have demonstrated that the pH of culture medium can significantly impact the antibacterial activity of RLs and the growth of certain *B. cereus* [17]. To further explore the pH adaptability of the experimental strain, the medium was monitored 24 h later in the initial pH range of 3.0 to 10.0 (Figure 1). It was observed that the medium was clear and transparent in the initial pH of 3.0 and 4.0, while the medium was turbid in the initial pH range from 5.0 to 10.0, indicating that *B. cereus* was able to grow and reproduce normally in the pH range from 5.0 to 10.0. It was documented that the RLs extracted from *Pseudomonas aeruginosa* OBP1 were effective against *Staphylococcus aureus* MTCC3160 and *Klebsiella pneumoniae* MTCC618 on an acidity scale ranging from 5 to 9, with the most pronounced inhibition zone being observed at pH 7 for both strains [20]. Subsequently, in accordance with the instructions for the purchased strains used in the experiment, *B. cereus* was cultured in the initial pH 7.0 medium for further experiments. 

### 2.2. Antibacterial Activity of RLs against B. cereus

**Figure 2:**
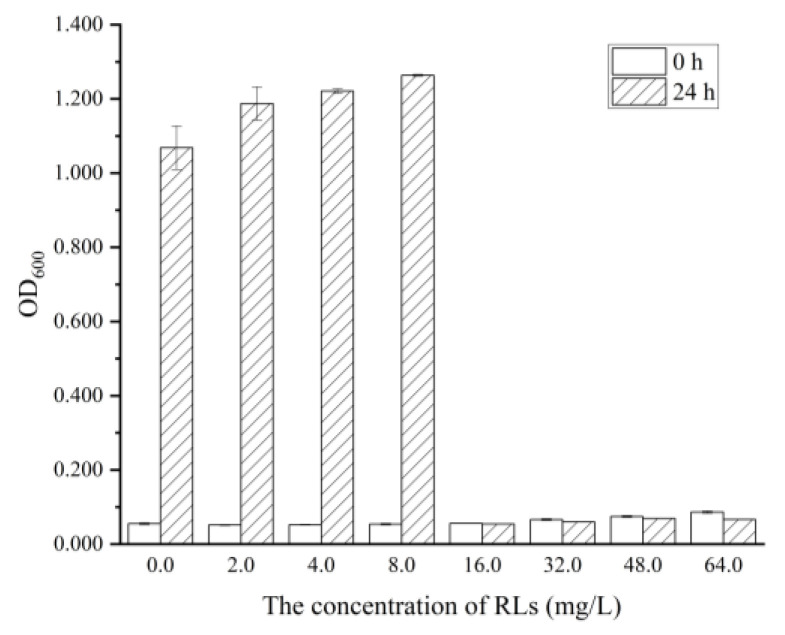
The Minimal Inhibitory Concentration (MIC) of RLs against *B. cereus*.

MIC and MBC are often used in the development of new antibacterial agents, as they can measure the inhibitory and lethal effects of the agents on particular microorganisms. The absorbance spectrophotometry and plate coating method were used to measure the MIC and MBC, respectively. As shown in Figure 2, the OD600 of the medium after 24 h of cultivation significantly increased (*p* < 0.05) with 0–8.0 mg/L RLs added, which indicated that low concentrations had no inhibitory effect on the growth of *B. cereus*. When the concentration of RLs reached 16.0 mg/L, there was no significant difference between the initial OD600 and OD600 after 24 h of cultivation. The MIC of RLs against *B. cereus* was determined to be 16.0 mg/L. As shown in Table 1, the total number of bacterial colonies in the control group was 5.50 ± 0.06 log CFU/mL. It was found that when the concentration of RLs was ≥32.0 mg/L, almost no bacterial colonies grew on the plate, indicating that the killing rate of 32.0 mg/L RLs on *B. cereus* was 99.9%. Therefore, the MBC of RLs against *B. cereus* was determined to be 32.0 mg/L. 

Recent research has revealed that a variety of eco-friendly substances, such as biosynthesis and plant extraction, possess antibacterial properties against *B. cereus*. Mannosylerythritol lipids, a type of glycolipid biosurfactant, have been found to demonstrate antibacterial activity against both vegetative cells and spores of Bacillus cereus, with a minimum inhibitory concentration (MIC) and minimum bactericidal concentration (MBC) of 1.25 mg/mL and 2.50 mg/mL, respectively [21]. Additionally, 4-[(4′-O-acetyl-α-L-rhamnosyloxy) benzyl] isothiocyanate (RBITC) derived from Moringa oleifera seeds was found to have an MIC and MBC of 0.14 mg/mL and 0.28 mg/mL, respectively [22]. These values were significantly higher than those of RLs in this study.

**Figure 3:**
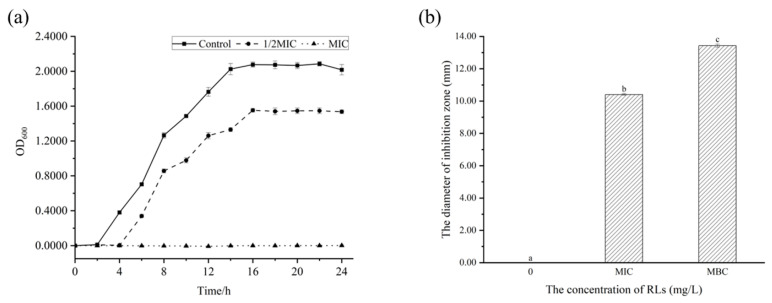
Effect of Rhamnolipids (RLs) on the growth of *B. cereus*. (**a**) Growth curve, (**b**) antibacterial zone. Significant difference (*p* < 0.05) was indicated by different letters.

To gain a more thorough understanding of the growth inhibition of RLs on *B. cereus* and the bactericidal effect of solid culture medium, the growth curve and antibacterial zone were examined. Figure 3 shows that *B. cereus* entered the logarithmic growth phase after 2 h and the stationary phase after 14 h. When 1/2 MIC concentration of RLs was added, the bacterial growth rate decreased with an extended delay period of 4 h, and the stationary phase began at 16 h, indicating that low concentrations of RLs can effectively slow the growth of *B. cereus*. Moreover, when MIC concentration of RLs was added, there was no turbidity present in the culture medium, suggesting complete inhibition of the bacteria’s growth, similar to the inhibition effect of RLs on *Listeria monocytogenes* [16]. The Oxford cup method was used to further confirm the antibacterial effect of RLs through an inhibition zone experiment. There was no inhibition zone in the control group, whereas inhibition zones of 10.41 ± 0.05 mm and 13.43 ± 0.10 mm were observed after treatment with RLs of MIC and MBC concentration, respectively. These results demonstrate the inhibitory effect of RLs on *B. cereus*.

### 2.3. Effect of RLs on the Morphology of B. cereus

**Figure 4:**
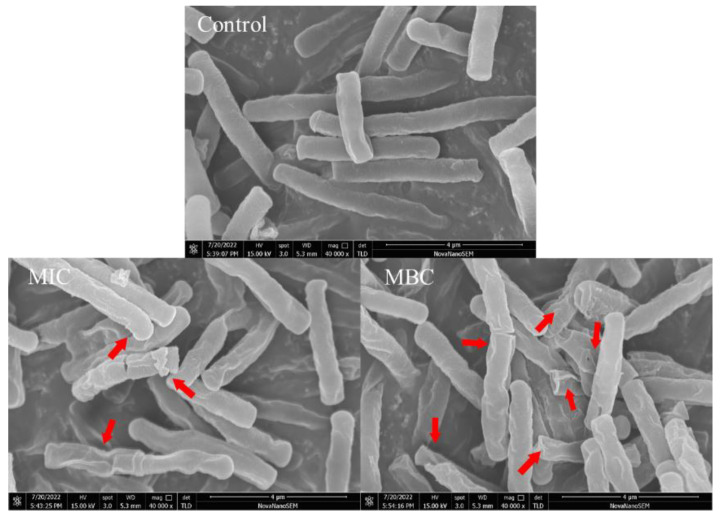
Scanning electron microscopy (SEM) images (×40,000) of *B. cereus* with different treatment. The red arrow marks the region of the *B. cereu* cell that has been damaged.

Scanning electron microscopy was used to observe the external morphological changes in *B. cereus* (Figure 4). Without the treatment of RLs, the cells of *B. cereus* were observed to maintain a rod-like shape, appearing plump with a smooth surface and no adhesion between them. However, after a 6 h treatment with RLs at an MIC concentration, the cells began to deform and exhibited an irregularly wrinkled surface and cell adhesion. When the concentration of RLs increased to MBC, the *B. cereus* cells were severely distorted with a folded and concave surface. The results suggested that RLs can disrupt the integrity of the *B. cereus* cell membrane, leading to decreased cell viability and death, which was the same in terms of the action to cell membrane between RLs and mannosylerythritol lipids [21].

The antimicrobial properties of rhamnolipids are ascribed to their amphiphilic character that allows them interaction with phospholipids, resulting in changes to the cytoplasmic membrane permeability and the loss of cell components, which ultimately leads to cell death [23]. Sana et al. observed that the roughness of bacterial cells was altered after treatment with biosurfactants, indicating that the sensitivity of bacteria to RL is connected to the capability to disrupt the cytoplasmic membrane, likely as a result of the disruption of the cytoplasmic membrane [24].

### 2.4. Effects of RLs on the Cell Membrane Permeability of B. cereus

The cell membrane is a critical component of cellular life systems, acting as a protective barrier to maintain cellular stability and facilitate nutrient transport. The integrity of the cell membrane was assessed by analyzing the ability of propidium iodide fluorescent dyes to enter cells, the membrane potential, the extracellular conductivity of the bacterial fluid and the release of intracellular macromolecules in this study.

**Figure 5:**
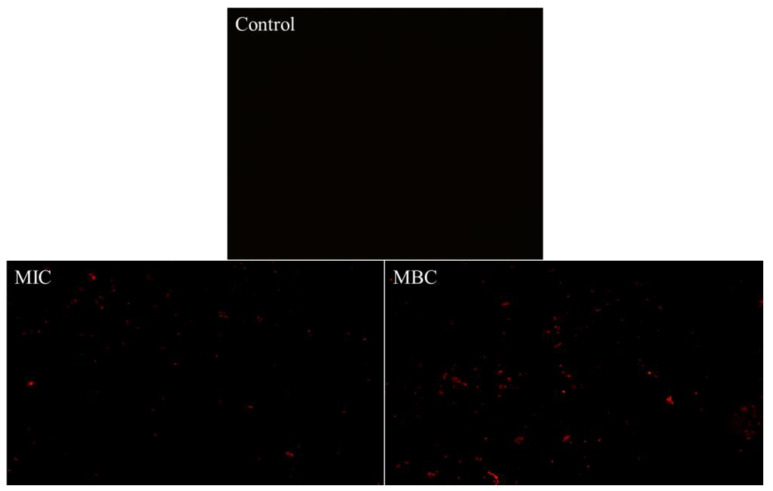
Fluorescence microscope images of *B. cereus* with different treatment.

Propidium iodide (PI) can enter the interior of cells through damaged cell membranes and selectively bind with DNA to generate fluorescent substances, so the stimulated red light can be observed through a fluorescence microscope to determine the integrity of the bacterial cell membrane [25]. Figure 5 demonstrates that the addition of RLs cause an increase in the red fluorescence of *B. cereus* when observed under a fluorescent microscope. The intensity of the stain was found to increase in a concentration-dependent manner as the concentration of added RLs increased from MIC to MBC, further indicating that the cell membrane of *B. cereus* was disrupted and its permeability enhanced.

**Figure 6:**
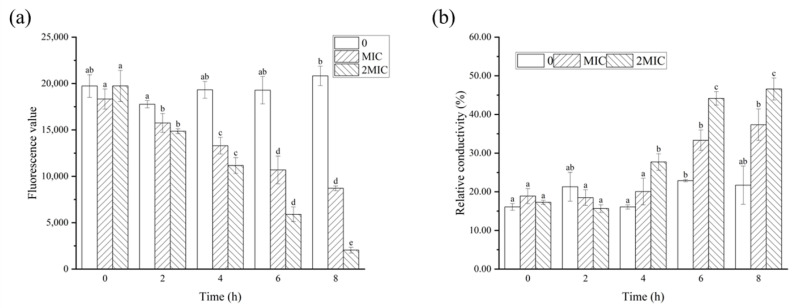
Effects of Rhamnolipids (RLs) on membrane potential (**a**) and relative conductivity (**b**) of *B. cereus*. Significant difference (*p* < 0.05) was indicated by different letters.

Meanwhile, the membrane potential and relative conductivity were measured to assess the cell membrane permeability, as shown in Figure 6. The membrane potential of *B. cereus* decreased significantly (*p* < 0.05) after RL treatment (Figure 6a), as indicated by the reduced fluorescence intensity of Rhodamine 123, a cationic fluorescence probe with cell membrane permeability [26]. This decrease in fluorescence intensity occurred due to the disruption of the cell membrane integrity and permeability, which led to the outflow of intracellular potassium ions and hyperpolarization of the cell membrane. This observation was supported by Han et al. [27], who discovered that the addition of juglone resulted in a depolarization of cell membranes, leading to abnormal metabolic activity. The relative conductivity of the control group showed no significant change over time (*p* > 0.05). However, the experimental group treated with RLs exhibited an increasing trend in the relative conductivity of the bacterial solution, with the MBC group showing a more pronounced increase (*p* < 0.05). After 8 h of treatment, the relative conductivity of the MBC group rose to 46.58%, which was twice that of the control group. This indicated that the permeability of *B. cereus* cell membrane was altered by RLs treatment, resulting in the leakage of electrolytes from the bacteria.

**Figure 7:**
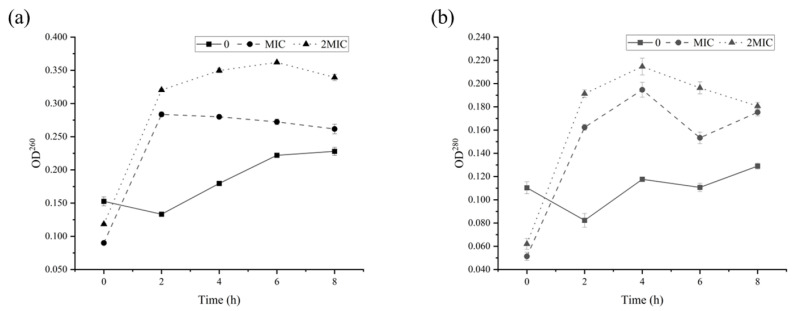
Release of nucleic acid (**a**) and protein (**b**) from *B. cereus* treated with Rhamnolipids (RLs).

Further, the integrity of the cell membrane can be assessed by analyzing the leakage of intracellular constituents including nucleic acids and proteins [28]. It has been observed that the OD_260_ and OD_280_ of *B. cereus* cells treated with RLs were significantly higher than those of the control group (*p* < 0.05), and the leakage of nucleic acid and protein in the MBC treatment group was higher than that in the MIC treatment group (Figure 7). These results suggest that RLs were able to disrupt the cell membrane of *B. cereus*, leading to the leakage of intracellular proteins, nucleic acids, and other macromolecules. 

### 2.5. Effects of RLs on the Oxidative Stress Response of B. cereus

Reactive oxygen species (ROS) are essential for normal physiological metabolism, as they are integral in maintaining redox homeostasis in the body. However, excessive production of highly oxidative reactive substances due to drug stimulation or insufficient scavenging ability of the body can lead to oxidative stress [29]. It has been demonstrated that even normal organisms produce ROS, with the body containing active oxygen enzymes that maintain a dynamic balance between oxidative damage and antioxidant defense [30]. The function of the enzyme SOD in living organisms is to utilize superoxide anions to dismutate ROS into hydrogen peroxide (H_2_O_2_) and oxygen (O_2_). H_2_O_2_ can then be broken down into water (H_2_O) and oxygen (O_2_) by the action of CAT [31].

**Figure 8:**
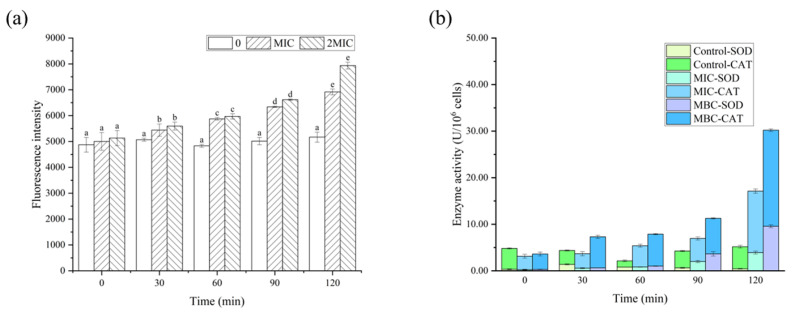
Effects of Rhamnolipids (RLs) on reactive oxygen species (a), superoxide dismutase (SOD) and catalase (CAT) activity (b) of B. cereus. Significant difference (p < 0.05) was indicated by different letters.

As demonstrated in Figure 8, the fluorescence values of the RL-treated group were significantly higher than those of the control, indicating that RLs caused a substantial amount of intracellular ROS production. ROS is known to be cytotoxic and can accumulate in large quantities, resulting in damage to bacterial biomolecules. The activities of CAT and SOD in *B. cereus* after being exposed to various concentrations of RLs for different durations were observed. This is likely due to the bacteria activating their oxidative stress response in response to RLs, which helps with chemical modification reactions taking place in proteins, lipids, and nucleic acids, altering their structure and function, thus having a detrimental effect on cell activity [29].

### 2.6. Effects of RLs on B. cereus in FWN

**Figure 9:**
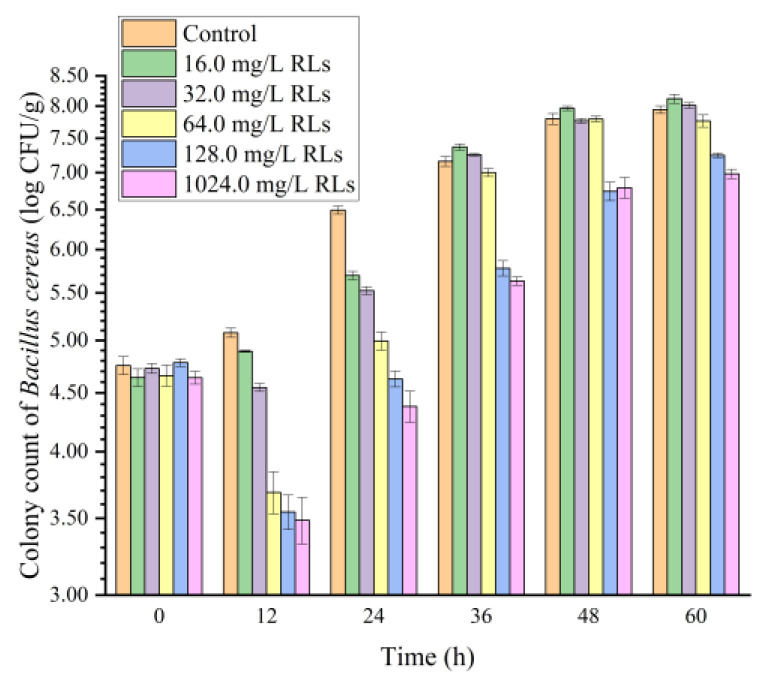
Effects of Rhamnolipids (RLs) on the growth of *B. cereus* in fresh wet noodles (FWN).

The antimicrobial potential of RL in controlling *B. cereus* using FWN as a food model was evaluated to study its applicability at the industrial level (Figure 9). Results showed that the addition of RLs (with concentrations of 16.0 mg/L and 32.0 mg/L) to FWN only inhibited the growth of *B. cereus* in the first 24 h. However, 16.0 mg/L RLs did not inhibit *B. cereus* in FWN over 36 h, and instead, the total number of colonies in the sample with 32.0 mg/L RLs reached the level of the control group. In contrast, the addition of RLs with concentrations of 64.0 mg/L, 128.0 mg/L and 1024.0 mg/L to FWN had a bactericidal effect on *B. cereus*, with an inhibition rate close to 100% in the 0–12 h stage. Furthermore, RLs with concentrations of 128.0 mg/L and 1024.0 mg/L reduced the number of bacteria over 1.0 log CFU/g, indicating the inhibition rate remained at about 90% during the entire storage period, which suggested that high concentrations of RLs can maintain the bactericidal effect in FWN for an extended period. Additionally, it was found that the concentration of RLs in FWN with significant bacteriostatic effect was significantly higher than the MIC in the culture medium, mainly due to the influence of food ingredients such as protein, starch, sugar and production process.

### 2.7. Effects of RLs on the Quality of FWN

#### 2.7.1. Color Changes in FWN

As the concentration of RLs increased, the color of FWN exhibited a slight fluctuation (Table 2). Nevertheless, all color values decreased over time, due to oxidation browning. However, the addition of RLs ranging from 16.0 to 1024.0 mg/kg did not have a significant effect on the colors of FWN (*p* > 0.05).

#### 2.7.2. Changes in the Moisture Content in FWN

**Figure 10:**
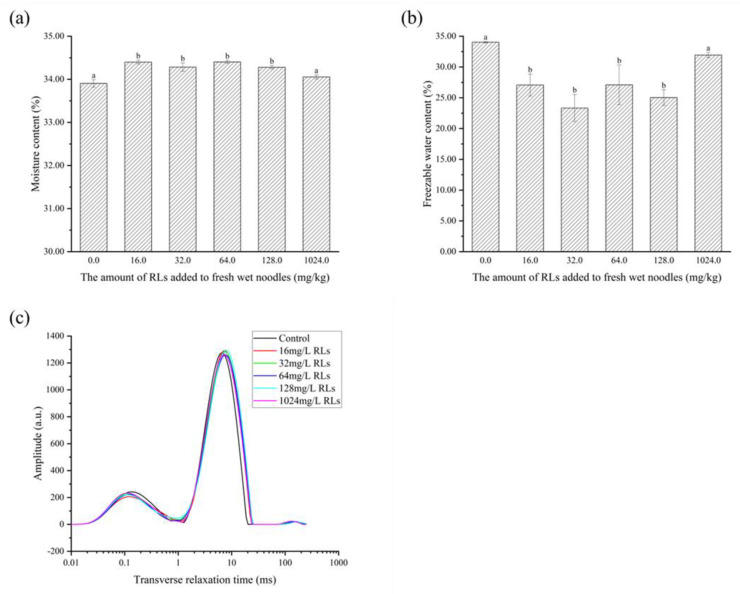
The alteration in total moisture content (**a**), freezable water (**b**) and relaxation time (**c**) of fresh wet noodles (FWN) treated with Rhamnolipids (RLs). Significant difference (*p* < 0.05) was indicated by different letters.

As shown in Figure 10a, the addition of RLs to FWN was found to increase the total water content due to its hydrating and hydrophilic properties [32]. Further analysis revealed that the freezable water content was significantly decreased (*p* < 0.05) in samples with RLs added (Figure 10b), likely due to RLs forming a closer bond with water, reducing the deep bound water activity and making it more difficult to freeze. This was further confirmed by the low-field nuclear magnetic resonance results (Figure 10c), which demonstrated that type I hydrogen proton peaks in fresh and wet surface samples with RLs shifted to the left, suggesting a stronger binding with water molecules. Thus, the addition of RLs from 16.0 to 1024.0 mg/L could reduce the free water content in FWN, thereby limiting the amount of water available to microorganisms and improving the storage stability of FWN.

#### 2.7.3. Alterations in the Rheology and Texture of FWN

**Figure 11:**
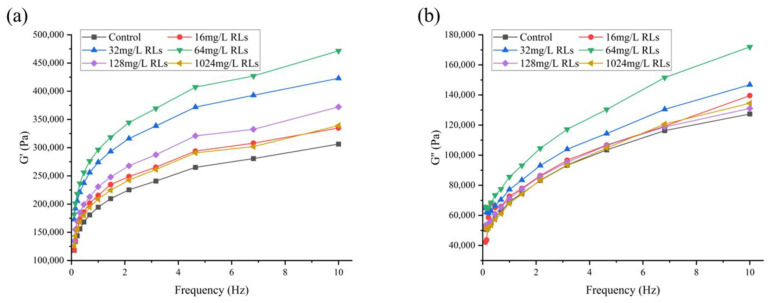
Changes in storage modulus G’ (**a**) and loss modulus G’’ (**b**) of fresh wet noodles (FWN) treated with Rhamnolipids (RLs).

The results depicted in Figure 11 show that the G’ and G’’ of fresh and wet noodle samples increase with the addition of RLs, which is likely because of the emulsifying effect of RLs that dominates the elasticity of the noodles. It is hypothesized that the hydrophobic tail of RLs binds to the hydrophobic sites of gluten proteins, counteracting electrostatic repulsion and thus resulting in a strengthened gluten network.

Texture Profile Analysis (TPA) is a common technique for gauging the quality of noodles, as it offers precision, repeatability, and quantification [33,34]. Table 3 indicates that the integration of RLs increased the hardness and chewiness of FWN, but had minimal influence on the elasticity and cohesion. Thus, RLs can improve the edible quality of FWN, making them more enjoyable to eat.

## 3. Materials and Methods

### 3.1. Strain and Culture Medium

The strain *Bacillus cereus* CMCC(B) 63301 was obtained from Beijing Jingbao Company (Beijing, China) and stored at −80 °C in nutrient broth culture medium supplemented with an equal-volume 50% glycerol. Nutrient broth culture medium consisted of peptone, 5.0 g; beef extract, 3.0 g; sodium chloride, 5.0 g; and distilled water, 1.0 L; pH adjusted to 7.0.

### 3.2. Reagents

RLs with a purity of over 90.0% were obtained from Huzhou Zijin Biotechnology Co., Ltd. (Huzhou, China) and stored at 25 °C. The RLs contained a small amount of water and soybean oil, which were residual from the fermentation process. The reactive oxygen species assay kit, the catalase (CAT) activity assay kit and the superoxide dismutase (SOD) activity assay kit were purchased from Beijing Solarbio Science and Technology Co., Ltd. (Beijing, China). Glucose and Rhodamine 123 were purchased from Shanghai Aladdin Biochemical Technology Co., Ltd. (Shanghai, China). Propidium iodide (PI) was purchased from Anhui Kule Biological Engineering Co., Ltd. (Hefei, China).

### 3.3. Effect of pH on the Growth of B. cereus

Nutrient broth culture medium was prepared according to the formula, and its pH was adjusted to 3.0, 4.0, 5.0, 6.0, 7.0, 8.0, 9.0, and 10.0 by using 1.00 mol/L hydrochloric acid and sodium hydroxide solutions, with each shake flask containing 50.0 mL. A 2% (*v/v*) bacterial suspension (OD_600_ was 1.2) was inoculated and incubated at 37 °C, 180 rpm for 24 h. Afterwards, OD_600_ of the bacterial suspension was measured.

### 3.4. Determination of Minimal Inhibitory Concentration (MIC) and Minimal Bactericidal Concentration (MBC)

The concentration of tested RLs ranged from 0 to 64.0 mg/L in an optimal pH medium with no RLs added as control. The medium was inoculated at 2% (*v/v*) and incubated at 37 °C, 180 r/min. The OD_600_ after 24 h was measured by a UV spectrophotometer (UV-2550, Shimadzu Corporation, Kyoto, Japan) and compared to the initial OD_600_. If the change in OD_600_ was less than 5%, the concentration was determined to be the MIC of RLs against *B. cereus*. A nutrient agar culture medium was prepared by combining peptone (5.0 g), beef extract (3.0 g), sodium chloride (5.0 g) and agar (20.0 g) in distilled water (1.0 L) and adjusting the pH to 7.0. Subsequently, the bacterial solutions cultured in the control group and each experimental group were diluted 100 times and 100 μL was applied to the 3 plates separately. The MBC was determined to be the lowest RL concentration in the experimental group that caused a 99.9% decrease in colony count (the minimum limit of detection was 10 CFU/mL), which was 3 orders of magnitude lower than the control group with no RLs added.

### 3.5. Characterization of Antibacterial Activity of RLs against B. cereus

RLs were added to the medium at concentrations of 0 (control), 1/2 MIC and MIC, and *B. cereus* was inoculated and incubated at 37 °C for 24 h. Bacterial growth was monitored at 600 nm using a UV spectrophotometer every 2 h, and the bacterial growth curve was plotted.

The Oxford cup experiment [35] was conducted by preparing RL solutions with MIC and MBC concentrations, with 100 µL of bacterial solution absorbed onto the solid medium and spread evenly. Empty sterile Oxford cups (Φ × φ × h, 7.8 mm × 6 mm × 10 mm) were positioned on the medium and 200 µL of RL solutions (including the control of pure culture medium) were added. The cups were incubated at 37 °C to check for a bacteriostatic zone and measure the size of the inhibition zone.

### 3.6. Observation of Cellular Morphology and Characterization of Intracellular Organization of B. cereus

#### 3.6.1. Scanning Electron Microscopy (SEM) Analysis

A *B. cereus* suspension with an OD_600_ of 1.2 was treated with RL concentrations of MIC and MBC, and incubated at 37 °C and 180 rpm for 6 h. The bacterial cells were then collected by centrifugation at 4 °C and 6000 rpm for 10 min, and subsequently washed three times with sterile 0.1 M PBS buffer (pH 7.2–7.4). The cells were fixed overnight with 2.5% glutaraldehyde before being dehydrated by successive gradients of 30%, 50%, 70% and 90% ethanol, followed by two washes with 100% ethanol. Subsequently, a mixture of ethanol and tert-butyl alcohol (2:1), ethanol and tert-butyl alcohol (1:1),and 100% tert-butyl alcohol was used to replace the ethanol and create a tert-butyl alcohol spore solution, which was dropped onto a pre-cooled aluminium foil at −80 °C and freeze dried. Following vacuum ion sputtering of the platinum film, the samples were then observed under a scanning electron microscope (SEM, Nova NanoSEM 450, FEI Company, Hillsboro, OR, USA) for analysis.

#### 3.6.2. Membrane Permeability Analysis

The medium was inoculated and incubated for 24 h at 37 °C, 180 r/min. Then, *B. cereus* suspensions were centrifuged at 4 °C, 8000 rpm for 10 min, followed by collection and washing with sterilized saline. Subsequently, RLs solutions with MIC and MBC concentrations were created, with the same volume of physiological saline as control, and 30 μmoL/L of PI was added. The mixture was kept away from light for 10 min and 3 μL of the liquid was placed under a fluorescent inverted microscope and photographed.

#### 3.6.3. Determination of Cell Membrane Potential

To evaluate the effect of RLs on the metabolic activity of bacteria, the cell membrane potential (MP) was determined by measuring the changes in the fluorescence intensity of Rhodamine 123 [36]. *B. cereus* suspensions (OD_600_ was 1.2) were prepared and exposed to 0 (control), MIC and MBC concentrations of RLs for 8 h. Samples were collected every 2 h, blended with rhodamine and incubated in the dark for 30 min. The fluorescence intensity was measured at an excitation wavelength of 480 nm and an emission wavelength of 530 nm through Multifunctional enzyme marker (SPARK, Tecan Corporation, Männedorf, Switzerland).

#### 3.6.4. Determination of the Electrical Conductivity

Bacterial suspensions (OD_600_ was 1.2) were centrifuged to obtain the bacterial cells, followed by washing with a 5% glucose solution, making sure that its conductivity was equal to that of the 5% glucose solution. Consequently, the bacterial suspension became an isotonic bacterial solution [37]. The conductivity was measured by adding 0 (control), MIC and MBC of RLs to a 5% glucose solution and recorded as L_1_. The isotonic bacteria were incubated for 8 h at 37 °C with shaking, and the supernatant conductivity was measured every 2 h, recorded as L_2_. The bacterial suspension cultured for 8 h was boiled in a water bath for 30 min, cooled and centrifuged, and the conductivity was recorded as L_0_. The relative electric conductivity of the bacterial solution was then calculated using the following Equation (1):

(1) $$\mathrm{Relative}\;\mathrm{electric}\;\mathrm{conductivity}\left(\%\right)=\frac{L_{2}-L_{1}}{L_{0}}\times \;100\%$$

#### 3.6.5. Determination of Released Intracellular Macromolecule

A total of 50.0 mL of the bacterial suspension (OD_600_ was 1.2) was taken, with the addition of different concentrations of RLs (MIC, MBC), supplemented with physiological saline as the control group. Then, the suspension was incubated at 37 °C, 180 rpm. At 0, 2, 4, 6 and 8 h, 2 mL of the bacterial suspension was collected and filtered through a 0.22 μm microporous membrane, after which the OD_260_ and OD_280_ of the filtrate were measured. 

#### 3.6.6. Determination of Reactive Oxygen Species (ROS)

Following centrifugation, collection and washing of the stable suspension with PBS three times, it was resuspended to 1 × 10^6^ CFU/mL. The RL solution was added to the suspension to reach the MIC and MBC concentrations before being incubated at 37 °C, 180 rpm. Samples of 200 uL were taken at 0, 30, 60, 90 and 120 min, and the probe was loaded according to the ROS assay kit instructions. The reaction was then conducted at 37 °C for 30 min. Afterwards, the suspension was centrifuged and washed with PBS. Finally, the fluorescence intensity was measured using an enzyme marker (SPARK, Tecan Corporation, Männedorf, Switzerland) at an excitation wavelength of 488 nm and an emission wavelength of 525 nm.

#### 3.6.7. CAT and SOD Enzyme Activities Analysis

The sample preparation process was the same as that described in Section 3.6.6, and the samples were incubated at 37 °C and 180 rpm, with samples taken at 0, 30, 60, 90 and 120 min. The samples were centrifuged at 4 °C and 8000 r/min for 10 min, followed by discarding the medium and washing 3 times with PBS. Subsequently, the absorbance values at 240 nm and 560 nm of samples were separately measured according to the instructions of the CAT and SOD activity assay kits.

### 3.7. Preparation of FWN

The preparation of FWN involved sample weighing, water addition, dough mixing, rolling, aging, secondary rolling, and strip cutting. To begin, 100.0 g of wheat flour was weighed separately and 30.0 mL of saline water containing 1.5 g of salt was added. The dough was then mixed for 5 min using a needle dough mixer. Next, the dough was rolled five times and placed in a self-sealing bag for 30 min to mature. Then, the dough was pressed once without folding on rolling pitches of 3, 2 and 1 mm respectively, followed by cutting into noodles with a thickness of 1.0 mm, a width of 2.0 mm and a length of 20 cm. Each batch of fresh wet noodles was made and used for each group of experiments, immediately.

### 3.8. The Inhibition of RLs against B. cereus in FWN

*B. cereus* suspension was sprayed onto the surface of noodles using a spray bottle to achieve a total of 4−5 log CFU/g. For the experimental group, the RL content in FWN was 16.0, 32.0, 64.0, 128.0 and 1024.0 mg/kg, respectively. FWN polluted *B. cereus* were packaged in preservation bags and stored at 25 °C with 25 g per bag separately for 24 h. In accordance with GB/T 4789.2-2010 National Standard for Food Safety Microbiological Examination, the total number of *B. cereus* was determined at 0 h, 12 h, 24 h, 36 h, 48 h and 60 h.

### 3.9. Assessment of the Quality of FWN

#### 3.9.1. Determination of the Colors of FWN

The color difference between FWN surfaces was determined using a color difference meter (CR-400, Hangzhou Ke Sheng Instrument, Hangzhou, China), which was represented by the L, a and b values.

#### 3.9.2. Determination of the Moisture Content and Freezable Water Content of FWN

The total moisture content of FWN was determined using the direct drying method in State Standard of the People’s Republic of China, the NO. of which is GB5009.3-2016.

A total of 10.0 mg of FWN was placed into an aluminum crucible, sealed and equilibrated for 3 min at 30 °C. Then, the sample was cooled from 30 °C to −20 °C at a rate of 10.00 °C/min, held at −20 °C for 5 min and heated from −20 °C to 10 °C at a rate of 1.00 °C/min. An empty crucible was used as a reference to calculate the freezable water content [38].

(2) $$FWC=\frac{∆\mathrm{Hfw}}{∆H\times w}$$

FWC is the content of freezeable water in the sample (%), ΔHfw is the melting enthalpy of fresh and wet surface (J/g), ΔH is the melting enthalpy of water (334 J/g), w is the moisture content of the sample (%).

#### 3.9.3. Determination of Water Molecule Migration

The surface was wrapped with 1.00 g of polytetrafluoroethylene (PTFE) tape and inserted into a 10 mm diameter nuclear magnetic tube, as suggested by Shi [39]. The nuclear magnetic tube containing the sample was situated in a permanent magnetic field of 0.05 T. The spin relaxation time T2 of the sample was detected using a Carr−Purcell−Meiboom−Gill (CPMG) pulse sequence at 32 ± 0.02 °C. The sampling frequency was set to 200 kHz, the RF delay was 0.020 ms, the waiting time was 2000 ms, the echo time was 0.100 ms, the number of echoes was 3000, and the number of repeated scans was 32. To obtain a T2 spectrum, 60,000 iterations were selected and the collected attenuation signal was inverted.

#### 3.9.4. Determination of Dynamic Rheology Properties

A 2 mm patch was placed on the test platform (diameter 35 mm) of the rotary rheometer (MARS iQ, Thermo Fisher Scientific, Waltham, MA, USA) to seal it; it was allowed relaxation on the parallel plate for 5 min, and its linear viscoelastic zone was measured under a stress of 50 Pa at a temperature of 25 °C and a frequency range of 0.10–10.00 Hz.

#### 3.9.5. Determination of the Texture

The steamed noodles were immediately taken out and rinsed with 25 °C water for 20 s. Any extra water from the surface was removed and the texture characteristics of the steamed noodles were measured by a texture analyzer (TA.XT plus, Stable Micro System, Surrey, UK) in a TPA mode using a probe model P36R. The TPA parameters were set to a pre-speed of 2 mm/s, a test speed of 1 mm/s, a post-test speed of 1 mm/s, a target mode of strain 50%, and a trigger force of 20.0 g.

### 3.10. Statistical Analysis

The data obtained in this study were expressed as the mean of three readings with the mean ± SD. Graphs were plotted using Origin 2022, and one-way analysis of variance (ANOVA) was conducted using SPSS 18.0, with a significance level of *p* < 0.05.

## 4. Conclusions

RLs exhibited inhibitory and inactivating effects on *B. cereus*, with a MIC of 16.0 mg/L and an MBC of 32.0 mg/L. RLs caused deformation of the morphogenesis of *B. cereus*, with folds and depressions on the surface and adhesion between cells. Moreover, RLs acted on the cell membrane, damaging its permeability and integrity, resulting in the leakage of intracellular macromolecules and metabolic disorder of *B. cereus*. Additionally, RLs caused a large accumulation of ROS in the bacteria, inducing oxidative stress reaction, damaging macromolecules in the bacteria, and affecting the growth and reproduction of *B. cereus*. RLs with additions of 128.0–1024.0 mg/kg were found to effectively inhibit the growth of *B. cereus* in FWN, maintaining an inhibition rate of around 90%, thereby improving the storage stability of FWN. In addition, RLs enhanced the edible quality of FWN, making it more resilient and chewy. The results suggested that RLs are expected to replace other chemical preservatives in food products.

## Figures and Tables

**Table 1:** The Minimal Bactericidal Concentration (MBC) of RLs against *B. cereus*.

RLs Content (mg/L)	Colony Count of *B. cereus* (log CFU/mL)	Killing Rate of *B. cereus* (%)
0 (Control)	5.50 ± 0.06 ^a^	0
2.0	5.12 ± 0.18 ^b^	56.59
4.0	5.29 ± 0.13 ^c^	37.45
8.0	4.78 ± 0.05 ^d^	81.17
16.0	3.33 ± 0.35 ^ab^	99.16
32.0	ND	≥99.90
48.0	ND	≥99.90
64.0	ND	≥99.90

Note: Significant difference (*p* < 0.05) was indicated by different letters in the table. “ND” stands for no colonies.

**Table 2:** The color of fresh wet noodles (FWN) treated with Rhamnolipids (RLs).

Color Value	RLs Content (mg/kg)	Storage Time/h					
		0	12	24	36	48	60
L	0 (Control)	76.65 ± 0.13 ^a^	76.05 ± 0.02 ^a^	76.03 ± 0.19 ^ab^	69.21 ± 0.30 ^a^	69.91 ± 0.85 ^bc^	67.39 ± 0.13 ^bc^
	16.0	76.17 ± 0.11 ^b^	76.44 ± 0.22 ^ab^	75.62 ± 0.36 ^bc^	69.30 ± 0.35 ^a^	71.52 ± 0.22 ^a^	70.57 ± 0.35 ^a^
	32.0	76.46 ± 0.18 ^ab^	76.58 ± 0.49 ^ab^	75.89 ± 0.27 ^abc^	68.28 ± 0.25 ^b^	70.36 ± 0.77 ^b^	67.61 ± 0.45 ^b^
	64.0	76.37 ± 0.30 ^ab^	76.85 ± 0.02 ^b^	76.26 ± 0.23 ^a^	69.22 ± 0.68 ^a^	69.55 ± 0.17 ^bc^	66.89 ± 0.08 ^c^
	128.0	76.16 ± 0.12 ^b^	76.71 ± 0.10 ^b^	75.49 ± 0.21 ^c^	68.33 ± 0.21 ^b^	68.45 ± 0.17 ^d^	67.38 ± 0.21 ^bc^
	1024.0	76.23 ± 0.13 ^b^	76.57 ± 0.32 ^ab^	75.63 ± 0.11 ^bc^	68.42 ± 0.10 ^b^	69.30 ± 0.19 ^cd^	67.73 ± 0.14 ^b^
a	0 (Control)	−0.68 ± 0.04 ^d^	−1.08 ± 0.04 ^a^	−0.63 ± 0.04 ^ab^	−1.89 ± 0.03 ^a^	−2.00 ± 0.01 ^b^	−2.01 ± 0.01 ^a^
	16.0	−0.65 ± 0.03 ^cd^	−0.75 ± 0.02 ^b^	−0.61 ± 0.06 ^ab^	−1.85 ± 0.04 ^a^	−2.08 ± 0.02 ^a^	−2.08 ± 0.03 ^b^
	32.0	−0.55 ± 0.07 ^b^	−0.56 ± 0.03 ^c^	−0.58 ± 0.05 ^b^	−1.85 ± 0.05 ^a^	−1.94 ± 0.04 ^c^	−2.01 ± 0.02 ^a^
	64.0	−0.54 ± 0.04 ^b^	−0.43 ± 0.03 ^d^	−0.67 ± 0.02 ^a^	−1.84 ± 0.03 ^a^	−2.02 ± 0.05 ^b^	−2.07 ± 0.04 ^b^
	128.0	−0.59 ± 0.04 ^bc^	−0.46 ± 0.12 ^cd^	−0.57 ± 0.03 ^b^	−1.84 ± 0.00 ^a^	−1.99 ± 0.02 ^bc^	−2.12 ± 0.03 ^b^
	1024.0	−0.44 ± 0.02 ^a^	−0.40 ± 0.05 ^d^	−0.47 ± 0.02 ^c^	−1.74 ± 0.01 ^b^	−1.80 ± 0.03 ^d^	−1.96 ± 0.02 ^a^
b	0 (Control)	18.36 ± 0.24 ^ab^	17.75 ± 0.15 ^ab^	18.44 ± 0.66 ^a^	11.70 ± 0.17 ^a^	11.92 ± 0.22 ^a^	11.43 ± 0.26 ^a^
	16.0	19.21 ± 0.36 ^a^	18.74 ± 0.10 ^c^	18.91 ± 0.13 ^a^	12.03 ± 0.15 ^a^	12.16 ± 0.14 ^ab^	11.35 ± 0.08 ^a^
	32.0	17.87 ± 0.09 ^b^	18.25 ± 0.34 ^abc^	18.75 ± 0.50 ^a^	11.76 ± 0.14 ^a^	11.57 ± 0.24 ^c^	10.79 ± 0.21 ^bc^
	64.0	17.79 ± 0.44 ^b^	17.70 ± 0.72 ^a^	18.90 ± 0.23 ^a^	11.80 ± 0.30 ^a^	11.94 ± 0.04 ^a^	10.73 ± 0.32 ^bc^
	128.0	18.22 ± 0.78 ^ab^	18.1 ± 0.29 ^abc^	18.75 ± 0.36 ^a^	12.03 ± 0.15 ^a^	12.29 ± 0.03 ^b^	10.57 ± 0.25 ^c^
	1024.0	18.36 ± 0.88 ^ab^	18.43 ± 0.10 ^bc^	19.29 ± 0.11 ^a^	11.99 ± 0.09 ^a^	12.11 ± 0.10 ^ab^	11.04 ± 0.05 ^ab^

Note: Significant difference (*p* < 0.05) was indicated by different letters in the table.

**Table 3:** The impact of Rhamnolipids (RLs) on the texture of fresh wet noodles (FWN).

RLs Content (mg/kg)	Hardness/g	Springiness	Cohesiveness	Chewiness	Resilience
0 (Control)	960.033 ± 27.75 ^e^	0.941 ± 0.005 ^a^	0.876 ± 0.005 ^a^	791.636 ± 26.201 ^a^	0.621 ± 0.011 ^a^
16.0	1008.009 ± 65.46 ^de^	0.943 ± 0.012 ^a^	0.859 ± 0.010 ^ab^	816.339 ± 49.019 ^ab^	0.610 ± 0.030 ^a^
32.0	1184.668 ± 22.21 ^a^	0.931 ± 0.012 ^a^	0.828 ± 0.014 ^bc^	912.874 ± 19.603 ^c^	0.546 ± 0.014 ^c^
64.0	1137.953 ± 24.80 ^ab^	0.939 ± 0.004 ^a^	0.833 ± 0.014 ^abc^	890.236 ± 2.617 ^bc^	0.569 ± 0.010 ^bc^
128.0	1105.841 ± 25.41 ^bc^	0.946 ± 0.010 ^a^	0.873 ± 0.013 ^a^	913.700 ± 33.171 ^c^	0.592 ± 0.017 ^ab^
1024.0	1049.238 ± 19.15 ^cd^	0.928 ± 0.018 ^a^	0.811 ± 0.049 ^c^	790.800 ± 66.200 ^a^	0.544 ± 0.008 ^c^

Note: Significant difference (*p* < 0.05) was indicated by different letters in the table.

## Data Availability

Data is contained within the article.

## References

[1] Franz C.M.A.P., den Besten H.M.W., Böhnlein C., Gareis M., Zwietering M.H., Fusco V. (2019). Reprint of: Microbial food safety in the 21st century: Emerging challenges and foodborne pathogenic bacteria. Trends Food Sci. Technol..

[2] Märtlbauer E., Granum P.E. (2021). *Bacillus cereus* Toxins. Toxins.

[3] Dietrich R., Jessberger N., Ehling-Schulz M., Märtlbauer E., Granum P.E. (2021). The Food Poisoning Toxins of *Bacillus cereus*. Toxins.

[4] López A.C., Minnaard J., Pérez P.F., Alippi A.M. (2015). A case of intoxication due to a highly cytotoxic *Bacillus cereus* strain isolated from cooked chicken. Food Microbiol..

[5] Takabe F., Oya M. (1976). An autopsy case of food poisoning associated with *Bacillus cereus*. Forensic Sci..

[6] Wijnands L.M., Dufrenne J.B., Rombouts F.M., Veld P.H.I.T., Leusden F.M.V. (2006). Prevalence of potentially pathogenic *Bacillus cereus* in food commodities in The Netherlands. J. Food Prot..

[7] Wu L.-m., Lai L., Lu Q., Mei P., Wang Y.-q., Cheng L., Liu Y. (2019). Comparative studies on the surface/interface properties and aggregation behavior of mono-rhamnolipid and di-rhamnolipid. Colloids Surf. B Biointerfaces.

[8] Hamzah N., Kasmuri N., Tao W., Singhal N., Padhye L., Swift S. (2020). Effect of rhamnolipid on the physicochemical properties and interaction of bacteria and fungi. Braz. J. Microbiol..

[9] Rocha V.A.L., de Castilho L.V.A., de Castro R.P.V., Teixeira D.B., Magalhaes A.V., Gomez J.G.C., Freire D.M.G. (2020). Comparison of mono-rhamnolipids and di-rhamnolipids on microbial enhanced oil recovery (MEOR) applications. Biotechnol. Prog..

[10] Irfan-Maqsood M., Seddiq-Shams M. (2014). Rhamnolipids: Well-Characterized Glycolipids with Potential Broad Applicability as Biosurfactants. Ind. Biotechnol..

[11] Costa S.G.V.A.O., Nitschke M., Haddad R., Eberlin M.N., Contiero J. (2006). Production of *Pseudomonas aeruginosa* LBI rhamnolipids following growth on Brazilian native oils. Process Biochem..

[12] Yang Y., Zhang M., Li J., Su Y., Gu L., Yang Y., Chang C. (2022). Construction of egg white protein particle and rhamnolipid based emulsion gels with β-sitosterol as gelation factor: The application in cookie. Food Hydrocoll..

[13] Kralova I., Sjöblom J. (2009). Surfactants Used in Food Industry: A Review. J. Dispers. Sci. Technol..

[14] Campos J.M., Stamford T.L., Sarubbo L.A., de Luna J.M., Rufino R.D., Banat I.M. (2013). Microbial biosurfactants as additives for food industries. Biotechnol. Prog..

[15] De Rienzo M.A.D., Stevenson P., Marchant R., Banat I.M. (2015). Antibacterial properties of biosurfactants against selected Gram-positive and negative bacteria. FEMS Microbiol. Lett..

[16] Magalhães L., Nitschke M. (2013). Antimicrobial activity of rhamnolipids against *Listeria monocytogenes* and their synergistic interaction with nisin. Food Control.

[17] Ferreira J.d.F., Vieira E.A., Nitschke M. (2019). The antibacterial activity of rhamnolipid biosurfactant is pH dependent. Food Res. Int..

[18] Bertuso P.d.C., Mayer D.M.D., Nitschke M. (2021). Combining Celery Oleoresin, Limonene and Rhamnolipid as New Strategy to Control Endospore-Forming *Bacillus cereus*. Foods.

[19] Bertuso P.d.C., Marangon C.A., Nitschke M. (2022). Susceptibility of Vegetative Cells and Endospores of *Bacillus cereus* to Rhamnolipid Biosurfactants and Their Potential Application in Dairy. Microorganisms.

[20] Bharali P., Saikia J.P., Ray A., Konwar B.K. (2013). Rhamnolipid (RL) from *Pseudomonas aeruginosa* OBP1: A novel chemotaxis and antibacterial agent. Colloids Surf. B Biointerfaces.

[21] Shu Q., Niu Y., Zhao W., Chen Q. (2019). Antibacterial activity and mannosylerythritol lipids against vegetative cells and spores of *Bacillus cereus*. Food Control.

[22] Shuangshuang W., Siyu L., Guo H., Lili Z., Xin L., Haiyan W., Long W., Jiaying Z., Wupeng G. (2022). Antimicrobial activity and mechanism of isothiocyanate from Moringa oleifera seeds against *Bacillus cereus* and *Cronobacter sakazakii* and its application in goat milk. Food Control.

[23] Sotirova A.V., Spasova D.I., Galabova D.N., Karpenko E., Shulga A. (2008). Rhamnolipid-biosurfactant permeabilizing effects on gram-positive and gram-negative bacterial strains. Curr. Microbiol..

[24] Sana S., Datta S., Biswas D., Sengupta D. (2018). Assessment of synergistic antibacterial activity of combined biosurfactants revealed by bacterial cell envelop damage. Biochim. Biophys. Acta (BBA)-Biomembr..

[25] Nescerecka A., Hammes F., Juhna T. (2016). A pipeline for developing and testing staining protocols for flow cytometry, demonstrated with SYBR Green I and propidium iodide viability staining. J. Microbiol. Methods.

[26] Mitchell P. (1966). Chemiosmotic coupling in oxidative and photosynthetic phosphorylation. Biol. Rev..

[27] Han Q., Feng L., Zhang Y., Zhang R., Wang G., Zhang Y. (2021). Effect of Juglone against *Pseudomonas syringae pv Actinidiae* Planktonic Growth and Biofilm Formation. Molecules.

[28] Kohanski M.A., Dwyer D.J., Collins J.J. (2010). How antibiotics kill bacteria: From targets to networks. Nat. Rev. Microbiol..

[29] Salehi F., Behboudi H., Kavoosi G., Ardestani S.K. (2018). Oxidative DNA damage induced by ROS-modulating agents with the ability to target DNA: A comparison of the biological characteristics of citrus pectin and apple pectin. Sci. Rep..

[30] Hao M., Liu R. (2019). Molecular mechanism of CAT and SOD activity change under MPA-CdTe quantum dots induced oxidative stress in the mouse primary hepatocytes. Spectrochim. Acta Part A Mol. Biomol. Spectrosc..

[31] Castro-Alférez M., Polo-López M.I., Marugán J., Ibáñez P.F. (2017). Mechanistic model of the *Escherichia coli* inactivation by solar disinfection based on the photo-generation of internal ROS and the photo-inactivation of enzymes: CAT and SOD. Chem. Eng. J..

[32] Eslami P., Hajfarajollah H., Bazsefidpar S. (2020). Recent advancements in the production of rhamnolipid biosurfactants by *Pseudomonas aeruginosa*. RSC Adv..

[33] Ajila C.M., Aalami M., Leelavathi K., Rao U.J.S.P. (2010). Mango peel powder: A potential source of antioxidant and dietary fiber in macaroni preparations. Innov. Food Sci. Emerg. Technol..

[34] Li M., Dhital S., Wei Y. (2017). Multilevel Structure of Wheat Starch and Its Relationship to Noodle Eating Qualities. Compr. Rev. Food Sci. Food Saf..

[35] Sun Q., Li C., Xu X., Zhao H., Liu C. (2021). Novel application of agarose in cultivating microorganisms in the stomach and rapid drug susceptibility testing of *Helicobacter pylori*. Mater. Express.

[36] Comas J., Vives-Rego J. (1997). Assessment of the effects of gramicidin, formaldehyde, and surfactants on *Escherichia coli* by flow cytometry using nucleic acid and membrane potential dyes. Cytometry.

[37] Zhang Y., Liu X., Wang Y., Jiang P., Quek S. (2016). Antibacterial activity and mechanism of cinnamon essential oil against *Escherichia coli* and *Staphylococcus aureus*. Food Control.

[38] Mi J., Liang Y., Lu Y., Tan C., Cui B. (2014). Influence of acetylated potato starch on the properties of dumpling wrapper. Ind. Crops Prod..

[39] Shi Z., Liu L., Zhang K., Wang X., Ma Z., Ren T., Li X., Xu B., Hu X. (2021). Effect of sheeting thickness on the processing quality of wheat-oat blended flour noodles. J. Cereal Sci..

